# The improvement of modified Si-Miao granule on hepatic insulin resistance and glycogen synthesis in type 2 diabetes mellitus involves the inhibition of TNF-α/JNK1/IRS-2 pathway: network pharmacology, molecular docking, and experimental validation

**DOI:** 10.1186/s13020-024-00997-9

**Published:** 2024-09-16

**Authors:** Zebiao Cao, Xianzhe Wang, Zhili Zeng, Zhaojun Yang, Yuping Lin, Lu Sun, Qiyun Lu, Guanjie Fan

**Affiliations:** 1 State Key Laboratory of Dampness Syndrome of Chinese Medicine, The Second Affiliated Hospital of Guangzhou University of Chinese Medicine & School of Basic Medical Sciences, Guangzhou University of Chinese Medicine, Guangzhou, China; 2 Department of Endocrinology, The Second Affiliated Hospital of Guangzhou University of Chinese Medicine, Guangzhou, China; 3 Postdoctoral Research Center, Guangdong Provincial Hospital of Chinese Medicine, Guangzhou, China; 4 Guangdong Provincial Academy of Chinese Medical Sciences, Guangzhou, China; 5 Guangzhou University of Chinese Medicine, Guangzhou, China; 6 Department of Breast Disease, The Second Affiliated Hospital of Guangzhou University of Chinese Medicine, Guangzhou, China; 7 School of Pharmaceutical Sciences, Guangzhou University of Chinese Medicine, Guangzhou, China

**Keywords:** Network pharmacology, Molecular docking, Modified Si-Miao granule, Berberine, Type 2 diabetes mellitus, Insulin resistance, TNF-α/JNK1/IRS-2 pathway

## Abstract

**Background:**

Modified Si-Miao granule (mSMG), a traditional Chinese medicine, is beneficial for T2DM and insulin resistance (IR), but the underlying mechanism remains unknown.

**Methods:**

Using network pharmacology, we screened the compounds of mSMG and identified its targets and pathway on hepatic IR in T2DM. Using molecular docking, we identified the affinity between the compounds and hub target TNF-α. Then these were verified in KK-Ay mice and HepG2 cells.

**Results:**

50 compounds and 170 targets of mSMG against IR in T2DM were screened, and 9 hub targets such as TNF and MAPK8 were identified. 170 targets were mainly enriched in insulin resistance and TNF pathway, so we speculated that mSMG might act on TNF-α, JNK1 and then regulate insulin signaling to mitigate IR. Experimental validation proved that mSMG ameliorated hyperglycemia, IR, and TNF-α, enhanced glucose consumption and glycogen synthesis, relieved the phosphorylation of JNK1 and IRS-2 (Ser^388^), and elevated the phosphorylation of Akt (Ser^473^) and GSK-3β (Ser^9^) and GLUT2 expression in KK-Ay mice. Molecular docking further showed berberine from mSMG had excellent binding capacity with TNF-α. Then, in vitro validation experiments, we found that 20% mSMG-MS or 50 μM berberine had little effect in IR-HepG2 cell viability, but significantly increased glucose consumption and glycogen synthesis and regulated TNF-α/JNK1/IRS-2 pathway.

**Conclusion:**

Network pharmacology and molecular docking help us predict potential mechanism of mSMG and further guide experimental validation. mSMG and its representative compound berberine improve hepatic IR and glycogen synthesis, and its mechanism may be related to the inhibition of TNF-α/JNK1/IRS-2 pathway.

**Supplementary Information:**

The online version contains supplementary material available at 10.1186/s13020-024-00997-9.

## Introduction

Type 2 diabetes mellitus (T2DM) is a metabolic disease characterized by hyperglycemia, insulin resistance (IR), and chronic inflammation [1]. According to the International Diabetes Federation Diabetes Atlas (10th), the number of diabetes patients worldwide reached 537 million (10.5%) in 2021, and T2DM accounts for about 90%; It is estimated that the total number of diabetes will increase to 643 million (11.3%) by 2030 and 783 million (12.2%) by 2045, which will pose a serious threat to global public health. Over the past decade, multiple hypoglycemic drugs have been launched, playing important roles in controlling blood glucose. However, these drugs are mostly single component, single target and do not conform to the complex pathological mechanisms of multiple causes of T2DM, and are often accompanied by certain adverse reactions [2]. Therefore, the increasing morbidity and total number of T2DM force us to find new effective prevention and control measures.

Complementary and alternative medicines, including traditional Chinese medicine (TCM), have become increasingly popular worldwide for treating sophisticated diseases such as cancer [3], diabetes [4], and non-alcoholic fatty liver disease (NAFLD) [5]. The Si-Miao formula, a classical TCM prescription composed of four herbs: *Atractylodis Rhizoma*, *Phellodendri Chinensis Cortex*, *Coicis Semen*, and *Achyranthis Bidentatae Radix*, has been recorded for clearing “dampness” and “heat” in “Cheng Fang Bian Du” by the prestigious physician Bingcheng Zhang in the Qing Dynasty [6]. Based on the TCM theory, “dampness” and “heat” are critical pathogenic factors leading to metabolic disorders such as obesity, NAFLD, and T2DM, which are characterized by IR [7]. Over the years, it has been widely used to treat diabetes and identified its anti-diabetic activity [7]. Recently, a study also found its anti-NAFLD effect [8]. In clinic practice, this prescription is often modified by replacing *Achyranthis Bidentatae Radix* with *Artemisiae Scopariae Herba* and *Plantaginis Herba* to increase its anti-inflammatory potency and hepatic targeting tendency, which is called modified Si-Miao granule (mSMG). Our previous clinical and experimental studies indicate that mSMG is beneficial for T2DM and IR [9–11]. However, its therapeutic mechanisms were largely unknown, warranting further investigations.

Chinese herbal formula consists of many different herbs, which may contain multiple active compounds and exhibit synergistic effects by acting on multiple targets of complex diseases with multiple causes [12]. However, this also makes the mechanism research of Chinese herbal formula very complicated and challenging. In recent years, with the development of system biology and bioinformatics, network pharmacology and molecular docking have emerged and become more directional and efficient method for the study of TCM by multiple scales of complicacy scope from compounds to targets [13, 14]. Using these methods, potential mechanisms of TCM formula have been elucidated in the treatment of complex diseases, such as HuangLian JieDu Decoction for sepsis [15], Qingfeiyin for acute lung injury [16], and Qizhu Tangshen formula for diabetic kidney disease [17].

**Fig. 1:**
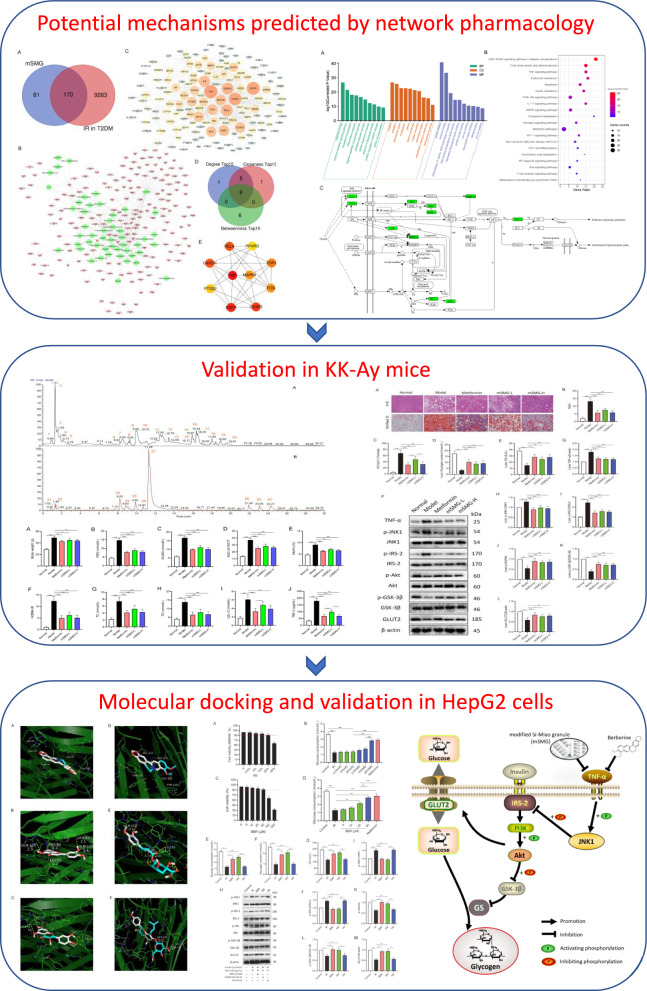
Workflow of the present study. We first used network pharmacology analysis to predict the compounds, action targets, and important signaling pathways of mSMG on reduction of IR in T2DM, and then combined with animal and cell experiments to verify the mechanism

In this study, based on network pharmacology, we identified hub targets TNF (Tumor necrosis factor, encodes TNF-α protein) and MAPK8 (Mitogen-activated protein kinase 8, encodes JNK1 protein) and key pathways, such as TNF pathway, MAPK pathway, and Phosphatidylinositol 3 kinase-Protein kinase B (PI3K–Akt) pathway. Molecular docking further showed berberine from mSMG had excellent binding capacity with the hub target TNF and might be involved in diabetes and insulin resistance. Therefore, we speculated that mSMG may act on the hub target TNF-α and JNK1 and then regulate insulin signaling to mitigate IR in T2DM. Experimental validation was conducted using KK-Ay T2DM mice and TNF-α-induced IR in HepG2 cells (IR-HepG2), and we confirmed that mSMG and its representative compound berberine could improve hepatic IR and glycogen synthesis, and its mechanism may be related to the inhibition of TNF-α/JNK1/IRS-2 pathway (Fig. 1). This study predicts underlying mechanism and displays scientific evidence for mSMG in the therapy of T2DM, which contributes to the drug development and may provide a new option for the complementary treatment of T2DM.

## Materials and methods

### Screening candidate compounds and targets of mSMG

As shown in Table 1, mSMG is a TCM formula consisting of five herbs: *Atractylodis Rhizoma* (AR, Pinyin name: Cangzhu, Plant source: *Atractylodes lancea* (Thunb.) DC.), *Phellodendri Chinensis Cortex* (PCC, Pinyin name: Huangbo, Plant source: *Phellodendron chinense* C.K.Schneid.), *Coicis Semen* (CS, Pinyin name: Yiyiren, Plant source: *Coix lacryma-jobi var. ma-yuen* (Rom.Caill.) Stapf), *Artemisiae Scopariae Herba* (ASH, Pinyin name: Yinchen, Plant source: *Artemisia capillaris* Thunb.), and *Plantaginis Herba* (PH, Pinyin name: Cheqiancao, Plant source: *Plantago asiatica* L.). The herbal name referred to the Chinese Pharmacopoeia 2020. Each herb corresponded to its plant source in the Chinese Pharmacopoeia 2020 and the latest revision of Medicinal Plant Names Services (MPNS, Royal Botanic Gardens, Kew, Version 12, accessed on September 13, 2023, http://mpns.kew.org). We screened the compounds of each herb in the Traditional Chinese Medicine Systems Pharmacology database (TCMSP, http://tcmspw.com/tcmsp.php) and Traditional Chinese Medicine integrative database (TCMID, http://119.3.41.228:8000/tcmid/) by entering the Pinyin names of herbs and filtered them with the screening criteria: oral bioavailability (OB) ≥ 30% and drug-likeness (DL) ≥ 0.18. The compounds from the TCMID database were imported into the TCMSP database due to no OB and DL parameters in the TCMID database, and only the compounds that met the screening criteria were included. The duplications of compounds were removed. Next, by entering the names of the screened compounds, corresponding targets were derived from TCMSP, Search Tool for Interacting Chemicals (STITCH, http://stitch.embl.de/), and DrugBank (https://www.drugbank.com/) databases. After deleting duplicate targets, their full names were input into the Uniprot database (https://www.uniprot.org/) and the National Center for Biotechnology Information (NCBI, https://www.ncbi.nlm.nih.gov/) for conversion into gene symbols.

**Table 1:** Information of mSMG

Pinyin name	Latin name	Plant source	Herb (g)	Granule: Herb	Granule (g)	Batch number of granule
Cangzhu	Atractylodis Rhizoma	*Atractylodes lancea* (Thunb.) DC.	10	1:7	1.43	21060040
Huangbo	Phellodendri Chinensis Cortex	*Phellodendron chinense* C.K.Schneid.	10	1:11	0.91	21010096
Yiyiren	Coicis Semen	*Artemisia capillaris* Thunb.	30	1:15	2.00	21100216
Yinchen	Artemisiae Scopariae Herba	*Coix lacryma-jobi var. ma-yuen* (Rom.Caill.) Stapf.	15	1:11	1.36	21070045
Cheqiancao	Plantaginis Herba	*Plantago asiatica* L.	30	1:12.5	2.40	21100012

### Screening genes related to IR in T2DM

The genes related to T2DM were collected by searching “Type 2 diabetes” or “Type 2 diabetes mellitus” or “Diabetes Mellitus, Non-Insulin-Dependent” or “T2DM” in the GeneCards (https://www.genecards.org/), OMIM (https://omim.org/) and DisGeNET (https://www.disgenet.org/) databases. Download files and delete duplicate genes. Meanwhile, the IR-associated genes were retrieved by searching “insulin resistance” in the three databases. Then, using the Draw Venn Diagram (http://bioinformatics.psb.ugent.be/webtools/Venn/), the overlaps between the genes related to T2DM and the IR-associated genes were filtered out, which were considered as the IR-associated genes in T2DM.

### Potential action targets of mSMG against IR in T2DM and compound-target network construction

In order to obtain the potential action targets, we used the candidate targets of mSMG to intersect the IR-associated genes in T2DM using the Draw Venn Diagram (http://bioinformatics.psb.ugent.be/webtools/Venn/). These overlapping targets and related candidate compounds were regarded as potential action targets and compounds of mSMG against IR in T2DM. Correspondingly, candidate compounds acting on these targets were also considered as potential compounds of mSMG against IR in T2DM. Subsequently, these potential compounds and action targets were used to construct the compound-target network and visualized by Cytoscape 3.7.1 software.

### Construction of PPI network and identification of hub targets

To explore the interaction among the potential targets of mSMG, we imported the potential action targets of mSMG against IR in T2DM into the Search Tool for the Retrieval of Interacting Genes/Proteins (STRING) database (https://cn.string-db.org/), selected “Homo sapiens” as the organisms, set high confidence (0.700) as a minimum required interaction score, and hided disconnected nodes in the network, then a protein–protein interaction (PPI) network was established. TSV format of the updated results were downloaded. Visualization and analysis were performed using Cytoscape 3.7.1. Firstly, node1, node2, and combined scores were extracted and imported into the Cytoscape software to create a PPI network, and the network was analyzed as follows: Step 1: analyze the topology properties of the network: Tools→Network Analyzer→Network Analysis→Analyze Network, save the CSV format of the network result and extract the Degree value; Step 2: create a network map according to the Degree value: Tools→Network Analyzer→Network Analysis→Generate Style from Statistics→Map Node Size to Degree (Low value to small sizes) →Map Node Color to Degree (Low value to dark colors), and save the PPI network map. Subsequently, we used 11 topological algorithms of plug-in cytoHubba [18] in Cytoscape 3.7.1 to identify the hub targets in the PPI network. Among the 11 methods, Degree, Closeness, and Betweenness are the most used to measure the importance of a node in a network. Degree refers to the number of nodes connected by a node; Closeness is used to measure the average distance between a node and other nodes; Betweenness is an indicator of node importance characterized by the number of shortest paths passing through a node [19]. The detailed operation process was as follows: Step 1, install cytoHubba: Apps→App Manager→Search cytoHubba→Install; Step 2: analyze the topological algorithms: cytoHubba→Calculate, save the CSV format of the calculation result and extract the Degree, Closeness, and Betweenness value. Afterwards, the top 15 targets respectively ranked by the 3 topological algorithms were intersected, and the overlapping targets were filtered out as the hub targets of mSMG against IR in T2DM.

### GO function and KEGG pathway enrichment analyses

To characterize the function and biological implications behind the potential action targets of mSMG, enrichment analyses of Gene Ontology (GO) and Kyoto Encyclopedia of Genes and Genomes (KEGG) pathway were carried out in the KEGG Orthology Based Annotation System database (KOBAS, http://kobas.cbi.pku.edu.cn/). We input gene symbols, selected “Homo sapiens (human)” as species, and set corrected *P*-value < 0.05 as statistical significance. Then, re-render the key KEGG pathway map using the “Pathview R” package. Based on the input action targets, add green color to the boxes corresponding to the relevant genes in KEGG pathway map to integrate information of KEGG pathway and action targets of mSMG against IR in T2DM for intuitive viewing.

### Reagents

Metformin (B25331) was purchased from Sino-US Shanghai Squibb Pharmaceutical Co., Ltd (Shanghai, China) and berberine (S9046) was purchased from Selleck (USA). TNF-α (11948), insulin receptor substrate 2 (IRS-2, 3089), Akt (4691), p-Akt (Ser^473^) (4060), glycogen synthase kinase-3β (GSK-3β, 12456), p-GSK-3β (Ser^9^) (5558), β-actin (8457), anti-rabbit IgG (7074), and anti-mouse IgG (7076) antibody were purchased from Cell Signaling Technology (Danvers, Massachusetts, USA). JNK1 (ab199380), p-JNK1 (T183) (ab47337), and glucose transporter 2 (GLUT2, ab54460) were purchased from Abcam (USA). p-IRS-2 (Ser^388^) (07-1517) was purchased from Sigma Aldrich Inc. Glucose kit (glucose oxidase method, A154-1-1), liver glycogen assay kit (A043-1-1), and enzyme linked immunosorbent assay (ELISA) kits for glycated hemoglobin (HbA1c, H464), insulin (H203), and TNF-α (H052) were purchased from Nanjing Jiancheng Bioengineering Institute (Jiangsu, China). Glycogen synthase activity assay kit (YB-GCS-1) was purchased from Shanghai Yubo Biotechnology Co., Ltd (Shanghai, China).

### Preparation and quality control of mSMG

All the herbs were processed into boil-free granules, provided by Sichuan Neo-Green Pharmaceutical Technology Development Co., Ltd (Sichuan, China) and quality controlled. Table 1 lists the amount of granular extracts of each herb in 1 unit of modified Si-Miao formula. They were mixed and brewed with boiling water like coffee based on Chinese Pharmacopoeia 2020. The main chemical components in mSMG were identified by Ultra-high-performance Liquid Chromatography-Quadrupole-Orbitrap Mass Spectrometry (UPLC-Q-Orbitrap-MS) as our previous description [20]. In brief, 1000 μL 80% methanol was added into 200 μL mSMG extract. Then it was swirled for 10 min, centrifuged at 4 °C for 10 min with centrifugal force of 20,000×*g*. Supernatant was filtered for UPLC-Q-Orbitrap-MS analysis. Mass spectrometric detection was performed on Q Exactive high resolution mass spectrometer (Thermo Fisher Scientific, USA). Mass spectrum condition: ion source: electric spray ionization source (ESI); scanning method: positive and negative ion switching scanning; detection method: full mass/dd-MS_2_; resolution ratio: 70,000 (full mass), 17,500 (dd-MS_2_); scan range: 100.0–1500.0 m/z; spary voltage: 3.2 kV(Positive, Negative); capillary temperature: 300 °C; collision gas: high purity argon (purity ≥ 99.999%); collision energy (N) CE: 30; sheath gas: nitrogen (purity ≥ 99.999%), 40 Arb; auxiliary gas: nitrogen (purity ≥ 99.999%), 15 Arb, 350 °C; data collection time: 30.0 min. Chromatographic analysis was performed on UltiMate 3000 RS (Thermo Fisher Scientific, USA). Chromatographic condition: chromatographic column: AQ-C18, 150 × 2.1 mm, 1.8 μm, Welch; flow rate: 0.30 mL/min; aqueous phase: 0.1% formic acid/aqueous solution; organic phase: methanol; column temperature: 35 °C automatic injector temperature: 10.0 °C; automatic injector volume: 5.00 μL. The data were preliminarily collated by CD2.1 (Thermo Fisher) and then retrieved and compared in mzCloud database.

### Animals and experimental design

Male C57BL/6J mice and KK-Ay mice aged 8 weeks were purchased from Beijing Huafukang Biotechnology Co., Ltd (Permission No: SCXK 2019–0008). All mice were individually housed in a specific pathogen-free (SPF) condition with free access to sterile water and chow diet. The housing ambient temperature was 22 ± 2 °C with a humidity of 55 ± 15% and a 12-h light/12-h dark cycle. All mice were allowed to acclimatize for 1 week. C57LB/6J mice fed with normal diet were taken as a normal control (Normal) group. KK-Ay mice were fed with high-fat diet (HFD; 4.73 kcal/g, 20% kcal from protein, 35% kcal from carbohydrates and 45% kcal from fat; H10045, Beijing Huafukang Biotechnology Co., Ltd, China). After 4 weeks, KK-Ay mice with fasting blood glucose (FBG) level more than 11.1 mmol/L were classified as T2DM [21], and then these mice were randomly divided into four groups (n = 6 per group): T2DM model group (Model), metformin treatment group (Metformin, 0.308 g/kg), low dose mSMG treatment group (mSMG-L, 1.665 g/kg), and high dose mSMG treatment group (mSMG-H, 3.330 g/kg). Dosage conversion between humans and mice was determined according to Pharmacological Experiment Methodology (4rd version) [22]. The dosages of metformin and mSMG in mice were respectively calculated by multiplying 0.025 g/kg (1.5 g metformin/60 kg adults/day) and 0.135 g/kg (8.1 g mSMG/60 kg adults/day) by 12.33 (conversion coefficient), resulting in an approximate dosage of 0.308 g/kg for metformin and 1.665 g/kg for the low dose mSMG, while the high dose was twice of that amount. mSMG suspension or metformin was administered to mice by oral gavage. Mice in Normal group and Model group were intragastric administered with the same solvent distilled water. Once a day. The adverse reactions were observed and recorded during the experiment. After 12 weeks of treatment, mice were euthanized, and blood and liver samples were collected. The animal protocols were strictly implemented following the Guidelines of the National Institutes of Health on Animal Care and Ethics and approved by the Animal Ethics Committee of Guangzhou University of Chinese Medicine (Approval Number: 20220512003).

### Oral glucose tolerance test

To detect the ability of the glycemic regulatory in mice, the oral glucose tolerance test (OGTT) was performed. After fasting for 15 h, glucose (1 g/kg) was administered by gavage, and blood glucose levels were measured using an automated analyzer (Roche Diagnostics, Germany) at 0, 30, 60, and 120 min, respectively. Then, the area under the curve (AUC) of blood glucose (AUC of OGTT) was calculated as following: AUC of OGTT = (G_0_ + G_120_)/4 + (G_30_ + G_60_)/4 + (G_60_ + G_120_)/2 [23].

### Serum biochemistry, HOMA-IR and TNF-α measurements

Blood samples were collected from the medial canthus vein and centrifuged (3000 r/min) for 15 min at 4 °C. FBG, total cholesterol (TC), triglyceride (TG), and low-density lipoprotein cholesterol (LDL-C), alanine aminotransferase (ALT), aspartate transaminase (AST), serum creatinine (Scr), and blood urea nitrogen (BUN) were examined by an automatic biochemistry analyzer (Rayto Technologies, China). HbA1c, fasting insulin (FINS), and TNF-α in serum were measured using ELISA kits according to the manufacturer’s protocol. Homeostasis model assessment-IR (HOMA-IR) was used to evaluate the IR in T2DM, which was calculated as described previously [23]: HOMA-IR = FBG (mmol/L) × FINS (μU/mL)/22.5.

### Hematoxylin and eosin staining

Briefly, after fixation in 4% paraformaldehyde for 24 h, liver tissues were isolated, dehydrated in graded volumes of ethanol alcohols, and embedded into paraffin. Then, the sections (3.5 μm) of liver tissues were stained by hematoxylin and eosin (H&E), and the pathology was observed under a microscope [21]. The H&E staining images were evaluated using the NAFLD activity score (NAS) system as previously reported [24].

### Oil Red O staining analysis

Frozen sectios (5 μm) of liver tissue were washed with phosphate buffered saline (PBS) twice. Then they were fixed in 4% paraformaldehyde for 40 min, washed twice in 60% isopropyl alcohol, and stained using an Oil Red O stain kit (P0047, Wuhan Pinofi Biotechnology Co., Ltd.) following the manufacturer’s instructions. Next, Positive cells were observed using an optical microscope [25].

### Molecular docking

To detect the binding capacity between compounds derived from the UPLC-Q-Orbitrap-MS analysis of mSMG and the hub target TNF identified by network pharmacology, we conducted molecular docking. Compounds of mSMG were selected as the ligands and the hub gene TNF was selected as the receptor. The molecule structures of ligands (compounds) were downloaded from the PubChem Database (https://pubchem.ncbi.nlm.nih.gov/). Based on the screening criteria: (1) protein source organism: Homo sapiens; (2) refinement resolution < 2.5 Å; (3) complete protein structure with corresponding ligand; and (4) pH within the physiological range of the human body, the molecule structures of receptor (2AZ5) were downloaded from the Protein Data Bank Database (www.rcsb.org). Then, we preprocessed the ligand using ChemBio3D software and automatically optimized its 3D conformation using the default system settings. PyMOL 1.7.2.1 software was utilized to remove water molecules and impurity from the receptor protein, followed by the separation of the original ligand to obtain a standardized receptor. Next, the ligand and receptor were imported into AutoDockTools 1.5.6 software. The receptor underwent the addition of polar hydrogen atom and Gasteiger charge. Subsequently, the Grid tool was utilized to automatically identify the Grid Box for the docking. The Grid Box parameters were set as follows: (1) Energy range and num modes were set as 5 and 20, respectively; (2) Spacing (angstrom) was 1; (3) The center of Grid Box was coincided with the center of the receptor; and (4) the dimension of Grid Box was manually adjusted based on the protein volume until the receptor was completely enveloped. Finally, molecular docking was performed using AutoDock Vina 4.2.6 software, and the docking results were visualized and assessed for hydrogen bond formation using PyMOL 1.7.2.1 software. Usually, binding energy lower than − 7.0 kcal/mol indicates excellent binding activity between receptor and ligand [15, 26].

### Preparation of mSMG-mediated serum (mSMG-MS)

Ten New Zealand rabbits were purchased from the Experimental Animal Center of Guangzhou University of Chinese Medicine and kept in a clean condition. The preparation and identification methods were the same as we described before [20]. In brief, the rabbits were randomly divided into the normal (n = 4) group and mSMG (n = 6) group. Rabbits in the mSMG group were administered mSMG 1.247 g/kg (three times the clinical equivalent dose) once a day, whereas animals in the normal group were intragastrically administered equal volume of distilled water. After continuous gavage for 6 days, the rabbits were fasted for 12 h. Then they were anaesthetized at 1 h after the last administration, and carotid blood samples were collected and centrifuged at 4000 r/min for 15 min. The supernatant was inactivated in a 56 °C water bath for 30 min and removed bacteria with a 0.22 μm microporous filter. Finally, we obtained the mSMG-mediated serum (mSMG-MS) and the normal serum (NS) and stored them at − 80 °C.

### Cell culture, TNF-α-induced IR model and treatment

The human hepatoblastoma cell line HepG2 was purchased from China Type Center for Type Culture Collection (CCTCC) (Wuhan, China) and cultured in Dulbecco’s Modified Eagle Medium (DMEM) containing 10% fetal bovine serum (FBS) at 37 °C in a humidified atmosphere containing 95% air and 5% CO_2_. According to the previous description [27, 28], IR in HepG2 cells (IR-HepG2 cells) was induced by 5 µM insulin and 30 ng/mL TNF-α for 4 h, and we treated the IR-HepG2 cells with corresponding drugs. Specifically, HepG2 cells were seeded in 6-well plates at a density of 1 × 10^4^ cells/well and grown for 48 h to 80–100% confluence. The cells were then incubated for 4 h in medium containing blank (Control group), or 5 µM insulin and 30 ng/mL TNF-α (IR group), or 5 µM insulin and 30 ng/mL TNF-α added with 20%NS (NS group) or 20% mSMG-MS (MS group) or 50 µM berberine (BBR group).

### Glucose consumption assay

Cellular glucose uptake was assayed in the medium with a glucose assay kit. After the treatment, culture medium was collected and separated at 1000 rpm for 10 min, and the supernatant was used to detect glucose content according to the manufacturer’s instructions. After incubation at 37 °C for 10 min, the absorbance was measured at 505 nm by a microplate reader (BioTek, Waltham, MA, USA) and the glucose content was calculated. Glucose consumption (mmol/L) = glucose content of blank well − glucose content of treatment well.

### Cell counting kit-8 (CCK-8) assay

The HepG2 cells (5 × 10^4^/well) were seeded in 96-well plates overnight. Then, the cells were incubated in medium with 5 μM insulin and 30 ng/ml TNF-α. Meanwhile, they were treated with mSMG-MS (0–40%) or berberine (0–250 µM) for 4 h. Next, 10 µL CCK-8 (C0038, Beyotime, China) was added to each well, and the cells were incubated on the condition of 37 °C, 5% CO_2_ for 2 h. Eventually, the absorbance was measured at 450 nm by a microplate reader, and cell viability (%) = optical density (OD) value of experimental group/OD value of control group × 100% [20].

### Glycogen content and glycogen synthase activity assay

After the treatment, glycogen was detected utilizing a glycogen assay kit according to the manufacturer’s instructions, and the absorbance was measured at 570 nm by a microplate reader. Additionally, the protein content was quantified by BCA assay kit (P0010, Beyotime, China), and the glycogen content was calculated as a ratio of glycogen (mg)/protein (g) [29]. Glycogen synthase (GS) is a key rate-limiting enzyme in glycogen synthesis. We measured it by ELISA method with a glycogen synthase activity assay kit according to the manufacturer’s instructions.

### Western blotting

According to our previously described method [20], the liver tissues or HepG2 cells were lysed with RIPA lysis buffer (P0013B, Beyotime, China) containing 1% protease inhibitor (5871, CST, USA) and 1% phosphatase inhibitor (5872, CST, USA). After quantification by BCA assay kit, the protein was separated by sodium dodecyl sulfate polyacrylamide gel electrophoresis (SDS-PAGE) and transferred to PVDF membrane. The PVDF membrane was sealed with 5% bovine serum albumin (BSA) on a shaker at room temperature for 1 h. Then, according to the molecular weight of the target protein, the PVDF membrane was incubated with TNF-α, JNK1, p-JNK1, IRS-2, p-IRS-2 (Ser^388^), Akt, p-Akt (Ser^473^), GSK-3β, p-GSK-3β (Ser^9^), GLUT2, or β-actin antibody on a shaker at 4 °C overnight. The next day, the membranes were incubated with species-related horseradish peroxidase (HRP)-conjugated secondary antibody (1:5000) at room temperature for 2 h. Finally, Ultra-sensitive enhanced chemiluminescent (ECL) (SI254786A, Thermo, USA) was employed and ChemiDoc™ XRS + System (Bio-Rad, USA) was used to display protein bands. Image J software (version 1.8.0, NIH, USA) was used to analyze the gray values.

### Statistical analysis

SPSS statistical program (version 25.0, IBM Corp, Armonk, USA) and GraphPad prism (version 8.0 La Jolla, CA, USA) were performed for statistical analyses and scientific graphing. When the data conformed to the normal distribution and the variance was homogeneous, the one-way Analysis of Variance (ANOVA) was used. When the one-way ANOVA had statistical differences, the Tukey test is used for multiple comparisons, and the data was described by the mean ± standard deviation ($\overline{x }$±*s*). When the data did not conform to the normal distribution or the variance is uneven, Nonparametric *Kruskal–Wallis H* test was used, and the data was described by M (*P25-P75*). All statistics were conducted by double-sided test. The test level (α) was 0.05, and the difference was statistically significant with *P* < 0.05.

## Results

### Candidate compounds and targets of mSMG

In TCMSP and TCMID databases, 73 compounds with OB ≥ 30% and DL ≥ 0.18 were obtained, such as worenine, berberine, and quercetin. There were 37, 13, 10, 9, and 9 compounds in *Phellodendri Chinensis Cortex*, *Artemisiae Scopariae Herba*, *Plantaginis Herba*, *Coicis Semen*, and *Atractylodis Rhizoma*, respectively. Some compounds were found in two or more herbs. Meanwhile, 231 targets of the compounds were searched in TCMSP, SITICH, and DrugBank databases.

### IR-associated genes in T2DM and action targets of mSMG against IR in T2DM

**Fig. 2:**
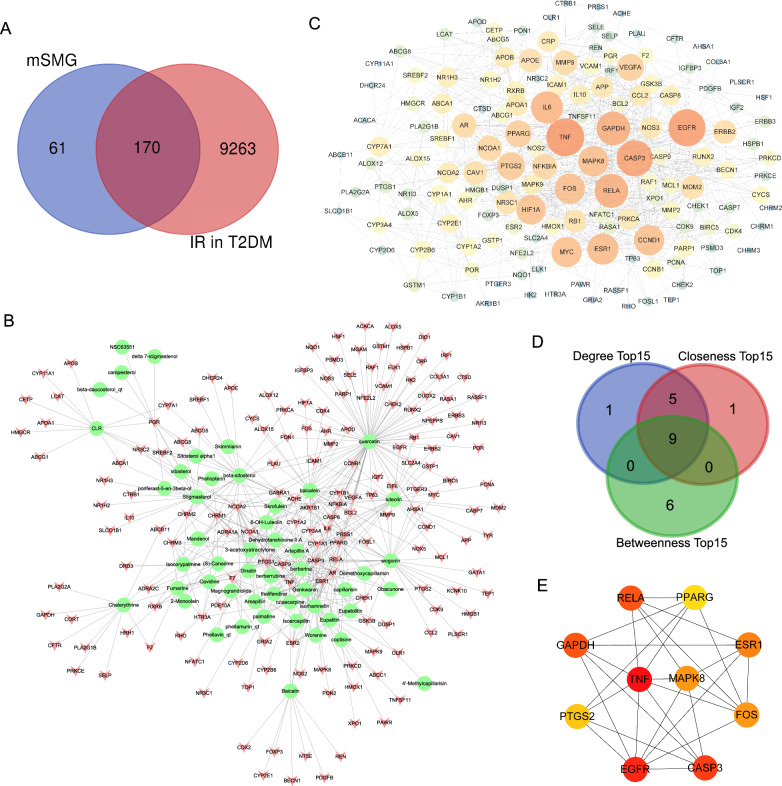
Potential action targets, compound-target network, PPI network, and hub targets of mSMG against IR in T2DM. **A** 170 intersecting targets between IR-associated genes in T2DM and candidate targets of mSMG were considered as the potential action targets of mSMG against IR in T2DM. **B** Compound-target network. A chartreuse ellipse represents a compound and a red “V” shape represents a target. **C** PPI network. A node represents a target. The size and the color of the node represents the value of the degree. Blue → yellow → red indicates that the degree value is from low to high, and the bigger the circle, the higher the degree value. **D** The intersecting 9 targets of the top 15 targets ranked by the 3 topological algorithms (Degree, Closeness, and Betweenness) were identified as the hub targets of mSMG against IR in T2DM. **E** PPI network of the 9 hub targets. The color of the node represents the value of the degree. Yellow → orange → red indicates that the degree value is from low to high

18,760 genes related to T2DM were collected in GeneCards, OMIM and DisGeNET databases. Meanwhile, 10,758 IR-associated genes were obtained in the three databases. Then, 9433 overlapping genes were filtered out by Venn diagram, which were considered as the IR-associated genes in T2DM patients. Furthermore, we took the intersection between IR-associated genes in T2DM and candidate targets of mSMG, and obtained 170 targets, which were considered as the action targets of mSMG against IR in T2DM (Fig. 2A).

### Compound-target network, PPI network and hub targets

Then, the compound-target network was constructed using the Cytoscape 3.7.1 software. As shown in Fig. 2B and Table S1, there were 50 compounds (such as quercetin, wogonin, baicalein, isorhamnetin, and berberine) and 170 targets (such as TNF, MAPK8, and GSK3B) in the network. It was seen that one compound could regulate multiple targets while one target could be controlled by multiple compounds, and mSMG might exert its efficacy through multiple compounds and multiple targets. Besides, the action targets of mSMG against IR in T2DM were input into the STRING database to PPI network, which contained 149 nodes and 852 edges after hiding the disconnected nodes (Fig. 2C). Next, the top 15 targets respectively ranked by Dgree, Closeness, and Betweenness were intersected, and we finally obtained nine targets, including TNF, MAPK8, FOS, RELA, PPARG, CASP3, PTGS2, EGFR, and GAPDH (Fig. 2D, E, Table 2), which were considered as the hub targets of mSMG against IR in T2DM.

**Table 2:** Nine hub targets identified by 3 topological algorithms in the PPI network

Target symbol	Encoded protein	Degree	Closeness	Betweenness
TNF	TNF-α	37	86.86667	1038.86446
EGFR	EGFR	36	86.78333	1405.93938
CASP3	CASP3	35	87.33333	1592.75902
RELA	NF-κB p65	32	84.66667	1298.52202
GAPDH	GAPDH	32	84.33333	1192.99561
FOS	c-Fos	28	81.83333	1020.78267
MAPK8	JNK1	28	81.03333	845.77760
PTGS2	COX-2	25	79.23333	1712.10864
PPARG	PPAR-γ	24	80.50000	976.27373

### Potential mechanisms of mSMG against IR in T2DM

**Fig. 3:**
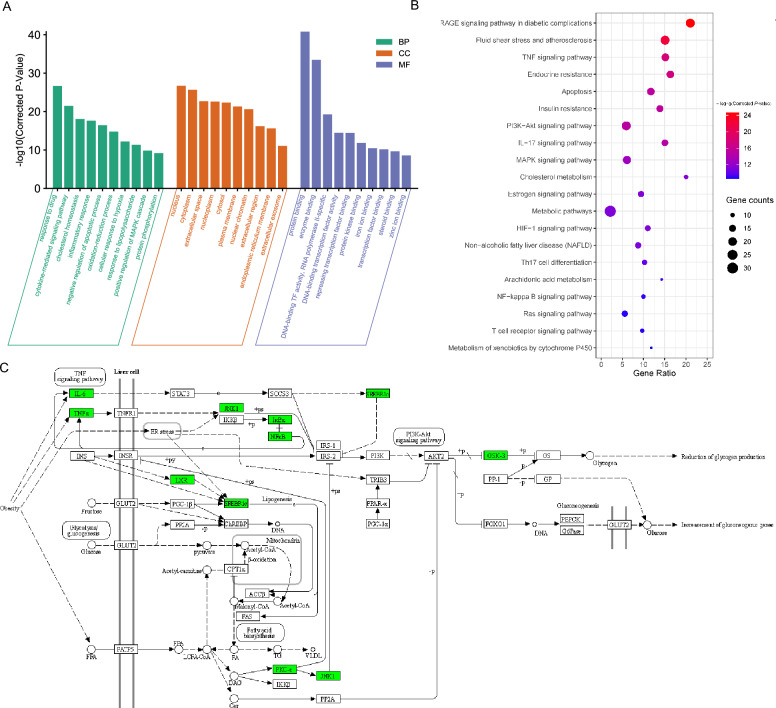
GO term and KEGG Pathway enrichment of the potential action targets of mSMG against IR in T2DM. **A** GO terms, including biological process (BP), cellular component (CC), and molecular function (MF). **B** KEGG Pathways. **C** KEGG Pathway map of Insulin resistance in liver cell in T2DM. The arrow (→) indicates promotion, and the T-arrows (⊣) indicates inhibition. The potential action targets of mSMG against IR in T2DM were marked as green color, and the raw map can be viewed at https://www.kegg.jp/kegg/mapper/

The action targets of mSMG against IR in T2DM were significantly enriched in 1,141 GO terms and 189 pathways, and the top 30 GO terms and top 20 KEGG pathways were presented in Fig. 3A, B. It could be seen that they were mainly concentrated in insulin resistance, inflammation, and metabolism (such as cholesterol homeostasis, inflammatory response, Fluid shear stress and atherosclerosis, TNF pathway, Insulin resistance, PI3K-Akt pathway, IL-17 pathway, MAPK pathway, and NF-κB pathway). KEGG Pathway map of Insulin resistance in liver cell was shown in Fig. 3C. From the map, we could see that the TNF-α/JNK1/IRS/PI3K/Akt pathway is the key mechanism in hepatic IR. Interestingly, the protein TNF-α and JNK1, encoded by the hub target TNF and MAPK8, respectively, were all enriched in this pathway. Therefore, we speculated that mSMG may act on these targets and then regulate insulin signaling pathway to alleviate IR in T2DM. Based on this scientific hypothesis, we conducted subsequent experimental verification.

### Compounds of mSMG based on UPLC-Q-Orbitrap-MS

**Fig. 4:**
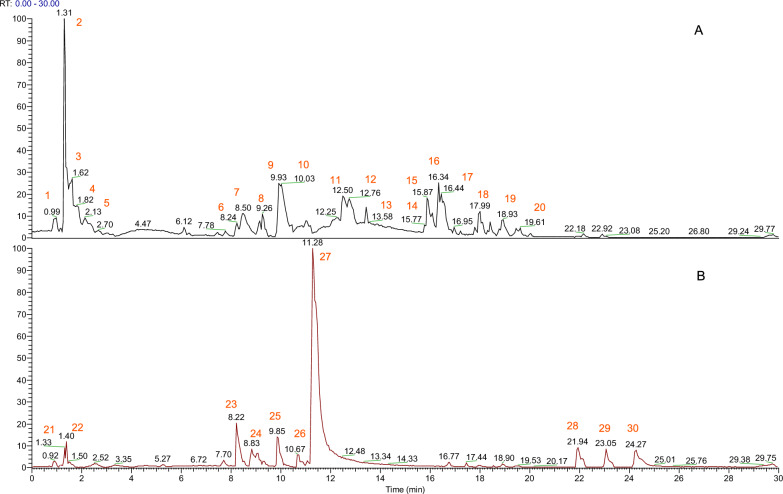
30 representative compounds of mSMG were identified based on UPLC-Q-Orbitrap-MS analysis. **A** Representative ion chromatogram of mSMG in the negative mode. **B** Representative ion chromatogram of mSMG in the positive mode

As shown in Fig. 4, the UPLC-Q-Orbitrap-MS analysis of mSMG was employed, and Table S2 showed that 30 compounds with higher abundance in mSMG, including berberine, luteolin, quercetin, wogonin, baicalein, coptisine, asperulosidic acid, and isorhamnetin, most of which were consistent with our prediction results by network pharmacology.

### mSMG ameliorates hyperglycemia, hyperlipidemia, IR and TNF-α without obvious adverse reactions in KK-Ay mice

**Fig. 5:**
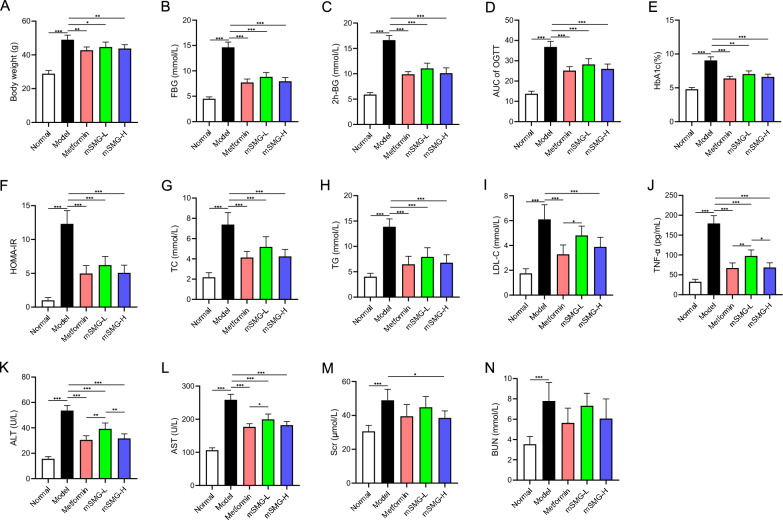
mSMG ameliorated hyperglycemia, hyperlipidemia, IR and serum TNF-α in KK-Ay mice. **A** Body weight. **B** Fasting blood glucose. **C** 2-h blood glucose of OGTT. **D** The area under the curve of OGTT. **E** HbA1c. **F** HOMA-IR. **G** Serum total cholesterol. **H** Serum triglyceride. **I** Serum low density lipoprotein cholesterol. **J** Serum tumor necrosis factor-α. **K** Alanine aminotransferase. **L** Aaspartate transaminase. **M** Serum creatinine. **N** Blood urea nitrogen. Data are expressed as mean ± SD (n = 6, *P < 0.05, **P < 0.01, ***P < 0.001)

First, we observed the effects of mSMG on blood glucose, blood lipids, insulin resistance and TNF-α. As shown in Fig. 5A–J, KK-Ay mice exhibited increased body weight, FBG, 2 h-BG, AUC of OGTT, HbA1c, TC, TG, LDL-L, HOMA-IR, and TNF-α, which were significantly reversed after mSMG treatment, especially in the high-dose group. Moreover, individual mice in the metformin group experienced transient but self-recovering diarrhea and anorexia during the initial treatment period, while no drug-related obvious adverse events were observed in mSMG-treated groups, such as diarrhea, hypoglycemia, and death. Compared to the model group, elevated ALT, AST, and Scr were significantly reduced after mSMG treatment, indicating that mSMG was not only safe, but could also alleviate liver and kidney function abnormalities induced by HFD in KK-Ay mice (Fig. 5K–N).

### mSMG improves liver histopathology in KK-Ay mice

**Fig. 6:**
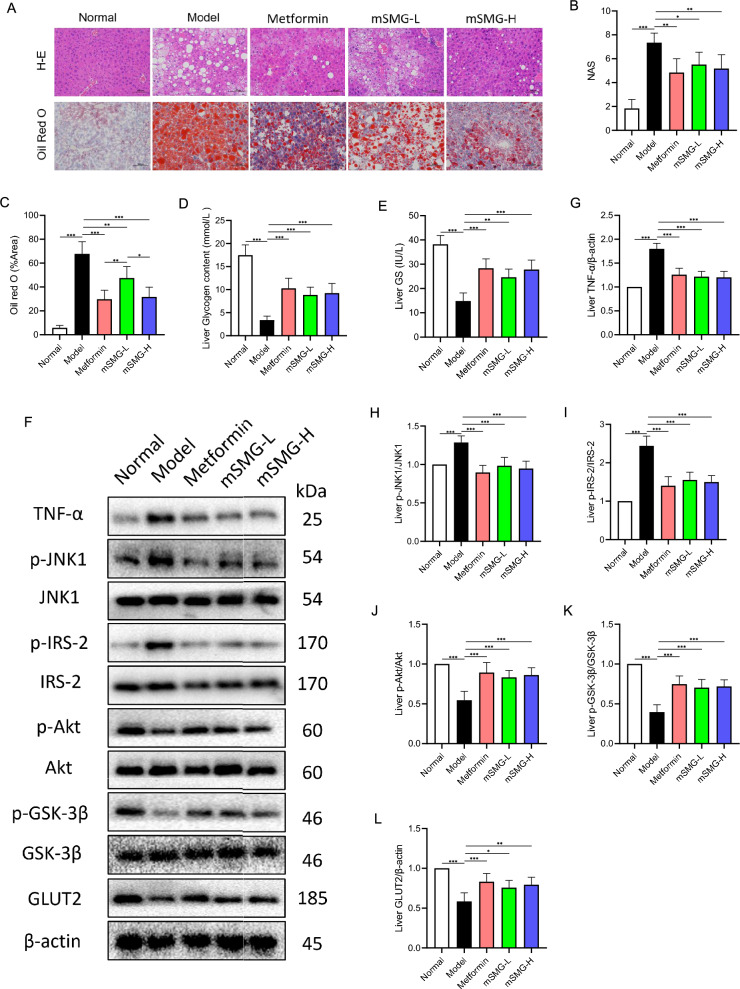
mSMG alleviated IR, promoted glycogen synthesis, and inhibited TNF-α/JNK1/IRS-2 pathway in liver of KK-Ay mice. **A** Representative H&E and Oil Red O staining images of liver sections (magnification 200×). **B** NAFLD activity score. **C** Positive area percentage of Oil Red O staining images. **D** Glycogen content in liver. **E** Glycogen synthase activity. **F**–**L** mSMG reduced the expression of TNF-α protein and the phosphorylation of JNK1 and IRS-2 (Ser^388^), and promoted the phosphorylation of Akt (Ser^473^) and GSK-3β (Ser^9^) as well as the expression of GLUT2 protein in liver. Data are expressed as mean ± SD (n = 6, *P < 0.05, **P < 0.01, ***P < 0.001)

H&E staining and Oil Red O staining analysis revealed that liver tissues of KK-Ay mice had obvious NAFLD characteristics with extensive fat vacuoles, liver cells balloon-like transformation, and interstitial inflammatory cell infiltration (Fig. 6A–C), which were consistent with the high serum TG, TC, LDL-C, ALT, AST, and TNF-α. Notably, these changes were mitigated by mSMG treatment, indicating that mSMG could repair NAFLD lesions in T2DM.

### mSMG promotes glycogen synthesis in the liver of KK-Ay mice

The liver is an important organ for glucose metabolism and homeostasis. Reduced glycogen synthesis is an important marker of hepatic IR, and glycogen synthase (GS) is a key rate-limiting enzyme in glycogen synthesis. We found the glycogen content and GS activity were significantly diminished in the liver of KK-Ay mice (Fig. 6D, E), which were accord with blood biochemical indicators and pathology, confirming significant insulin resistance in the liver of KK-Ay mice. However, mSMG markedly enhanced the GS activity and glycogen synthesis (Fig. 6D, E).

### mSMG inhibits hepatic TNF-α/JNK1/IRS-2 pathway in KK-Ay mice

Based on the predicted results of network pharmacology, we studied the action mechanism of mSMG from TNF-α/JNK1/IRS-2 pathway. We found that proinflammatory factor TNF-α in the Model group was significantly higher than that in the Normal group. Compared with the Model group, TNF-α was significantly less in mSMG group, no matter in the serum or liver, indicating that mSMG could reduce systemic and local TNF-α (Fig. 5J, Fig. 6F, G). Furthermore, we found significant changes of key proteins in TNF-α/JNK1/IRS-2 pathway and insulin signaling. The phosphorylation of JNK1 and IRS-2 (Ser^388^) were significantly upregulated, whereas the phosphorylation of Akt (Ser^473^) and GSK-3β (Ser^9^) as well as the expression of GLUT2 protein were observably downregulated in the liver of KK-Ay mice (Fig. 6F, H–L). Nevertheless, mSMG treatment significantly restored the levels of these proteins (Fig. 6F, H–L). These results suggest that the mechanism by which mSMG reduces hepatic IR and promotes glycogen synthesis may be related to the inhibition of TNF-α/JNK1/IRS-2 pathway. We further confirmed this through in vitro experiments.

### Molecular docking

**Fig. 7:**
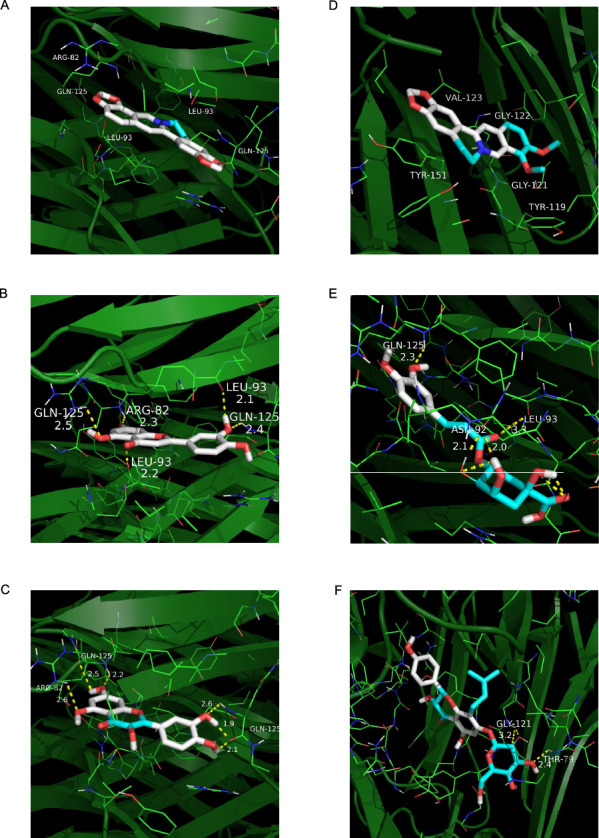
Molecular docking between the hub target TNF-α and the top 6 compounds from mSMG ranked by affinity. Molecular docking between TNF-α and **A** coptisine, **B** luteolin, **C** quercetin, **D** berberine, **E** palmatine, and **F** chlorogenic acid, respectively. The label represents the amino acid compositions around the ligand, the yellow line represents the hydrogen bond, and the number next to the yellow line represents the length of the hydrogen bond

We ranked the molecules in ascending order according to binding energy and chose the top 10 molecules in the results to show in Table 3. The results showed that no compound’s binding energy was higher than 0 kcal/mol and the average affinity between 30 compounds and TNF-α is − 6.95 kcal/mol, which indicates potentially spontaneous binding capacity between these compounds and TNF-α. Certainly, 16 compounds’ binding energy with TNF-α is lower than − 7.0 kcal/mol and only 4 compounds’ binding energy is higher than − 5.0 kcal/mol. Those compounds showing excellent binding activity were majorly derived from *Phellodendri Chinensis Cortex*, *Artemisiae Scopariae Herba*, and *Plantaginis Herba*. Then, the docking structures of the top 6 compounds ranked by affinity were visualized. As showed in Fig. 7B, luteolin formed five hydrogen bonds with Gln125, Arg82, and Leu93, and quercetin formed five hydrogen bonds with Gln125 and one hydrogen bond with Arg82 in TNF-α (Fig. 7C). Palmatine formed two hydrogen bonds with Asn92, one hydrogen bond with Leu93, and one hydrogen bond with Gln125 in TNF-α (Fig. 7E). Figure 7F showed that chlorogenic acid interacted with Gly121 and Thr79 of TNF-α through one hydrogen bond separately. Figure 7A and D show the docking outcomes between TNF-α and coptisine as well as berberine, respectively, although they did not form any hydrogen bonds with TNF-α. Generally, the involvement of hydrogen bonds was not necessarily required in molecular docking, and other intermolecular interaction forces, such as van der Waals forces and electrostatic interactions, can have significant impacts on the bonding between molecules. The high affinities between these two molecules and TNF-α support the presumption that they bind solidly. In short, the potential binding possibilities between the compounds from mSMG and TNF-α were displayed by molecular docking. Interestingly, combining the results of molecular docking and previous reports, we found that among the top 10 compounds ranked by affinity in molecular docking, berberine was reported to be more involved in diabetes and insulin resistance. Therefore, we subsequently conducted in vitro experiments with berberine as a representative component.

**Table 3:** Top 10 compounds ranked by affinity with TNF-α in molecular dockings

Compounds	PubChem ID	Affinity (kcal/mol)	Structural classification	Source
Coptisine	72322	− 9.3	Alkaloids	*Phellodendri Chinensis Cortex*
Luteolin	5280445	− 9.0	Flavonoids	*Plantaginis Herba*
Quercetin	5280343	− 8.9	Flavonoids	*Phellodendri Chinensis Cortex*, *Artemisiae Scopariae Herba*
Berberine	2353	− 8.8	Alkaloids	*Phellodendri Chinensis Cortex*
Palmatine	19009	− 8.8	Alkaloids	*Phellodendri Chinensis Cortex*
Chlorogenic acid	1794427	− 8.7	Phenylpropanoids	*Artemisiae Scopariae Herba*, *Plantaginis Herba*
Cafestol	108052	− 8.6	Terpenoids	*Phellodendri Chinensis Cortex*
Phellamurin	193876	− 8.5	Flavonoids	*Phellodendri Chinensis Cortex*
baicalein	5281605	− 8.4	Flavonoids	*Plantaginis Herba*
Arcapillin	158311	− 8.2	Flavonoids	*Artemisiae Scopariae Herba*

### Effect of mSMG-MS and berberine on cell viability and glucose consumption in IR-HepG2 cells

**Fig. 8:**
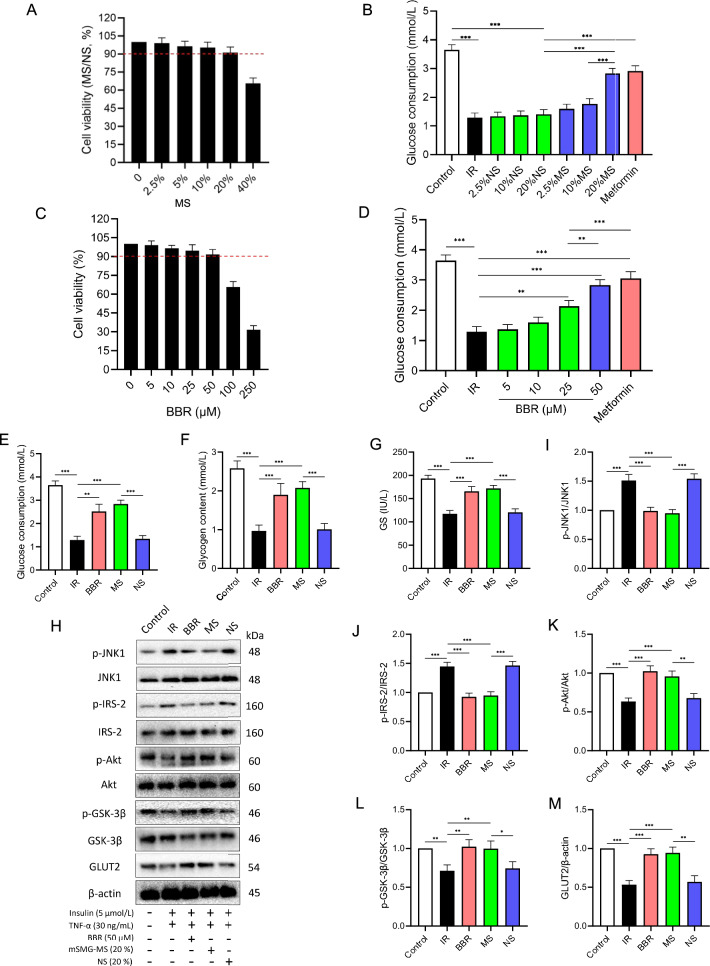
mSMG-MS or berberine alleviated IR and promoted glycogen synthesis in IR-HepG2 cells, which may be related to the inhibition of TNF-α/JNK1/IRS-2 pathway. **A** Cell viability after treatment with different concentrations of mSMG-MS (0–40%) or berberine (0–250 μM) in IR-HepG2 cells for 4 h. **B** Glucose consumption of HepG2 cells after treatment with 5 μM insulin and 30 ng/mL TNF-α (IR), or 5 μM insulin and 30 ng/mL TNF-α added with 0.5 mM metformin or mSMG-MS (2.5–20%), or berberine (5–50 μM) for 4 h. 20% mSMG-MS or 50 μM berberine (**C**–**E**) improved glucose consumption, glycogen content, and glycogen synthase activity, **F**–**H** decreased the phosphorylation of JNK1 and IRS-2 (Ser^388^), and **F**, **I**–**K** increased phosphorylation of Akt (Ser^473^) and GSK-3β (Ser^9^) as well as the expression of GLUT2 protein in TNF-α-induced IR HepG2 cells. HepG2 cells were incubated for 4 h in medium containing blank (Control group), or 5 μM insulin and 30 ng/mL TNF-α (IR group), or 5 μM insulin and 30 ng/mL TNF-α added with 20%NS (NS group) or 20%MS (MS group) or 50 μM berberine (BBR group). Data are expressed as mean ± SD (n = 3, *P < 0.05, **P < 0.01, ***P < 0.001)

The glucose consumption is the net result of glucose uptake and efflux by cells, directly reflecting the utilization of exogenous glucose. First, based on the IR-HepG2 cells model, the cytotoxicity of mSMG-MS and berberine was analyzed, and the effect of mSMG-MS and berberine on glucose consumption was measured. As shown in Fig. 8A, when the concentration of mSMG-MS was 0%-20%, the survival rates of IR-HepG2 cells were more than 90%, indicating that mSMG-MS had no or little cytotoxicity to IR-HepG2 cells within this concentration range. Then we selected 2.5%, 10%, and 20% mSMG-MS to incubate IR-HepG2 cells for 4 h, respectively. As shown in Fig. 8B, the three doses of mSMG-MS increased the glucose consumption of IR-HepG2 cells in a dose-dependent way. Among them, the glucose consumption in the 20% mSMG-MS group was significantly higher than that in others, and there was no significant difference with the metformin group (0.5 mM), indicating that 20% mSMG-MS could effectively improve the utilization of exogenous glucose in IR-HepG2 cells. Similarly, 0–50 μM berberine had no or little cytotoxicity to IR-HepG2 cells and 25–50 μM berberine increased the glucose consumption of IR-HepG2 cells in a dose-dependent way (Fig. 8C). Among them, the glucose consumption at the concentration of 50 μM was significantly higher than that in others, and there was no significant difference with the metformin group, indicating that 50 μM berberine could effectively improve the utilization of exogenous glucose in IR-HepG2 cells (Fig. 8D). Therefore, 20% mSMG-MS and 50 μM berberine were selected as the intervention in the subsequent experiments.

### mSMG-MS and berberine enhance glycogen synthesis in IR-HepG2 cells

When IR occurs, in addition to reduced glucose uptake, glycogen synthesis in liver cells is hindered, which is also an important cause for hyperglycemia. As shown in Fig. 8E–G, the glucose consumption, glycogen content, and GS activity in the IR or NS group were significantly lower than those in the Control group. However, they were significantly increased by 20% mSMG-MS or 50 μM berberine, indicating that mSMG-MS and berberine could promote glucose transport and glycogen synthesis in IR-HepG2 cells (Fig. 7C–E).

### mSMG-MS and berberine reduce IR and promote glycogen synthesis by inhibiting TNF-α/JNK1/IRS-2 pathway in IR-HepG2 cells

Furthermore, we found significant changes in TNF-α/JNK1/IRS-2 pathway and insulin signaling. IR-HepG2 cells showed increased phosphorylation of JNK1 and IRS-2 (Ser^388^), and decreased phosphorylation of Akt (Ser^473^) and GSK-3β (Ser^9^) as well as the expression of GLUT2 protein, whereas 20% mSMG-MS or 50 μM berberine treatment significantly restored the levels of these proteins (Fig. 8H–M). These results suggest that the improvement of mSMG and berberine on hepatic IR and glycogen synthesis in T2DM involves the inhibition of TNF-α/JNK1/IRS-2 pathway, and berberine may be a representative compound of mSMG.

## Discussion

**Fig. 9:**
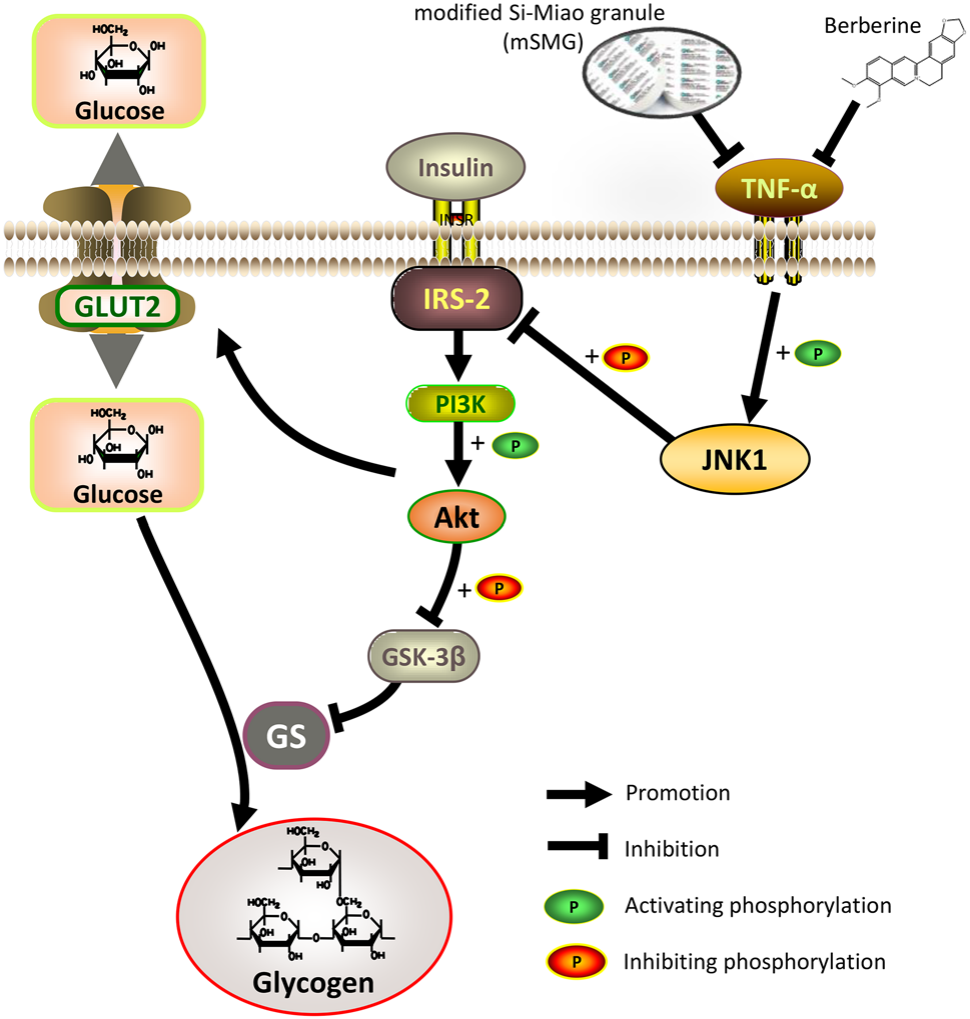
The biological mechanism of mSMG against hepatic IR in T2DM may involve the inhibition of TNF-α/JNK1/IRS-2 pathway. Elevated TNF-α promotes JNK1 phosphorylation, which phosphorylates serine sites of IRS-2 and then inactivate IRS-2, followed by the suppression of PI3K/Akt pathway. Subsequently, reduced Akt phosphorylation downregulates the GLUT2 levels and the phosphorylation of GSK-3β (Ser^9^), which activates GSK-3β and then leads to inhibition of GS activity, resulting in impaired glucose uptake and glycogen synthesis, known as hepatic IR. mSMG and its representative compound berberine may reverse this process by inhibiting TNF-α/JNK1/IRS-2 pathway

In this study, based on network pharmacology, 50 compounds and 170 action targets of mSMG against IR in T2DM were screened, and 9 hub targets such as TNF (encodes TNF-α protein) and MAPK8 (encodes JNK1 protein) were identified. These 170 action targets were mainly enriched in insulin resistance, inflammation, and metabolism, such as TNF pathway, MAPK pathway, and PI3K-Akt pathway. Molecular docking further showed berberine from mSMG had excellent binding capacity with TNF-α and might be involved in diabetes and insulin resistance. Experimental validation was conducted using KK-Ay T2DM mice and IR-HepG2 cells, and we confirmed that mSMG and its representative compounds berberine could ameliorate hepatic IR and promote glycogen synthesis, and its mechanism may be related to the inhibition of TNF-α/JNK1/IRS-2 pathway (Fig. 9). To the best of our knowledge, there have been no reports on the mechanism by which Si-Miao formula or its modified formula improve hepatic IR and glycogen synthesis based on TNF-α/JNK1/IRS-2 pathway. In addition, we not only conventionally conducted network pharmacology analysis and experimental verification in vitro and in vivo for mSMG, but also increased molecular docking to predict representative compound and further experiments to confirm its representativeness, which also has certain characteristics and novelty.

Due to the complex composition of TCM, studying its molecular mechanisms through animal or cell experiments requires a significant amount of manpower, material resources, and time [30]. Network pharmacology and molecular docking can systematically and efficiently reveal the multi-component network regulation and compound-target binding situation of TCM [5]. They have been widely integrated to explore compounds, action targets, and therapeutic mechanisms of TCM such as HuangLian JieDu Decoction [15], Qingfeiyin [16], and Qizhu Tangshen formula [17]. In this study, using the network pharmacology analysis, we identified 50 compounds and 170 action targets of mSMG on reduction of IR in T2DM, and found that it might act on the hub target TNF (encodes TNF-α protein) and MAPK8 (encodes JNK1 protein) to regulate TNF pathway, MAPK pathway, and PI3K-Akt pathway, thereby ameliorating IR in T2DM.

In T2DM, high levels of glucose and inflammatory cytokines can activate MAPK. JNK, one of the MAPK family, regulate several key serine sites, which are important negative regulatory factors in insulin signaling, thereby restraining insulin signaling and inducing IR [27]. Among them, the serine phosphorylation of IRS by JNK is closely related to IR. IRS is the key protein mediating insulin action, mainly including IRS-1, IRS-2, IRS-3, IRS-4. Among them, IRS-2 is highly expressed in liver and pancreatic islet β cells and affects liver glucose metabolism and the growth and differentiation of β cells [31, 32]. IRS-2 can promote PI3K activation and phosphorylated Akt [33]. JNK activation phosphorylate IRS at multiple serine sites, which diminishes its ability to undergo tyrosine phosphorylation and may accelerate the degradation of IRS, followed by reduced phosphorylation of Akt (Ser^473^) and GSK-3β (Ser^9^) [34], which leads to the decreased phosphorylation of GS, thus activating GS and promoting glycogen synthesis [35]. TNF-α is one of the most important pro-inflammatory mediators and plays a crucial role in the development of IR and pathogenesis of T2DM [36, 37]. It can promote JNK phosphorylation, which inhibits IRS-2/PI3K/Akt/GSK-3β pathway, resulting in impaired glucose uptake and glycogen synthesis [27, 28, 38]. Anti-TNF-α treatment strategies have been developed to reduce the incidence of insulin resistance [39]. Interestingly, our study found that the protein TNF-α and JNK1, encoded by the hub target TNF and MAPK8, respectively, were all enriched in TNF-α/JNK1/IRS/PI3K/Akt pathway. Therefore, we speculated that mSMG may act on the hub target TNF-α and JNK1 and regulate the insulin signaling to mitigate IR in T2DM. Subsequent experimental verification proved that mSMG could ameliorate hyperglycemia, IR, and serum and liver TNF-α. Besides, mSMG and mSMG-MS significantly enhanced glucose consumption, GS activity, and glycogen synthesis, relieved the phosphorylation of JNK1 and IRS-2 (Ser^388^) proteins, and elevated the phosphorylation of Akt (Ser^473^) and GSK-3β (Ser^9^) as well as the expression of GLUT2 protein in KK-Ay T2DM mice and IR-HepG2 cells. This is different from the conventional insulin sensitizer thiazolidinedione (activation of peroxisome proliferators-activated receptor γ) [40] and metformin (mainly through the activation of AMP-activated protein kinase) [41]. Moreover, our study showed that mSMG had fewer adverse reactions, and even reduced the deterioration of liver and kidney function induced by diabetes. In addition, a study also reported that modified Si-Miao-San extract inhibited inflammatory response and modulated insulin sensitivity through inhibiting NF-κB pathway and IRS-1 serine 307 phosphorylation and increasing downstream Akt (T308) activation in HepG2 cells stimulated with palmitate [7]. Similarly, network pharmacology analysis in this study also discovered that its hub target RELA (NF-κB subunit) and enriched pathway involved NF-κB pathway, and experimental verification found that mSMG alleviated TNF-α. Differently, our study focused on the downstream JNK pathway, IRS-2 and GLUT2 (main IRS and GLUT in the liver), as well as Akt (Ser^473^), and we found that mSMG and mSMG-MS inhibited the phosphorylation of IRS-2 (Ser^388^) and promoted the phosphorylation of Akt (Ser^473^) and the expression of GLUT2 in KK-Ay mice and TNF-α-induced IR-HepG2 cells. These suggest that mSMG alleviate hepatic IR and promote glycogen synthesis, and its mechanism may involve the inhibition of TNF-α/JNK1/IRS-2 pathway.

Molecular docking is a theoretical simulation technique used to validate intermolecular interactions and predict their binding capacity indirectly [15]. Our results showed the potential binding possibilities between the compounds from mSMG and TNF-α due to a low average binding energy (− 6.95 kcal/mol). Interestingly, the docking between TNF-α and berberine did not form any hydrogen bonds. Generally, the involvement of hydrogen bonds was not necessarily required in molecular docking, and other intermolecular interaction forces, such as van der Waals forces and electrostatic interactions, can have significant impacts on the bonding between molecules [16]. The high affinities between berberine and TNF-α support the presumption that they bind solidly. Combining the results of molecular docking and previous reports, we found that berberine was reported to be more involved in diabetes and insulin resistance among the top 10 compounds ranked by affinity. Therefore, we subsequently conducted in vitro experiments with berberine as a representative component. Consistent with the effect of mSMG, experiments verified that berberine also significantly stimulated glucose consumption, GS activity, and glycogen synthesis, repressed the phosphorylation of JNK1 and IRS-2 (Ser^388^) proteins, and facilitated the phosphorylation of Akt (Ser^473^) and GSK-3β (Ser^9^) as well as the expression of GLUT2 protein in IR-HepG2 cells. These results suggest that berberine may be a representative component of mSMG in anti-diabetes and anti-hepatic IR. There have been many reports on the improvement of berberine on hepatic IR by anti-inflammatory mechanisms, such as inhibiting IKK/NF-κB, JNK, and IRS-1/AKT pathways in gestational diabetes mellitus rats [42], attenuating ERK1/2-induced serine phosphorylation of IRS-1 and thus enhancing Akt (T308) activation in primary hepatocytes pre-treated with TNF-α [43], and inhibiting LPS/TLR4/TNF-α signaling in HFD-fed obese rats [44]. Coincidentally, this study also proved that berberine exert its anti-hepatic IR effect by modulating TNF-α, JNK pathway, and Akt pathway. Moreover, we further found that it is related to restraining the phosphorylation of IRS-2 (Ser^388^) and promoting the phosphorylation of Akt (Ser^473^) and GSK-3 β (Ser^9^) proteins as well as the expression of GLUT2 protein in TNF-α-induced IR-HepG2 cells.

It should be pointed out that there are still some limitations to this study. Firstly, the reliability and accuracy of predictions depend on data quality, and methods of searching compounds and targets from public literature and databases cannot exclude the existence of bias. Secondly, pharmacokinetic studies and quantitative analysis on the compounds should be conducted to determine the bioavailability of mSMG. Thirdly, considering the characteristics of multi-compound, multi-target, and multi-pathway of TCM, the effects of other compounds with low binding energy (such as coptisine, quercetin, chlorogenic acid) on hepatic IR and TNF-α/JNK1/IRS-2 pathway, and the effects of mSMG on other targets and pathways (such as RELA, PPARG, and IL-17 pathway) should be further explored in future. In addition, selecting other ages, genders, and housing conditions of animals for further experiments may help with more comprehensive validation.

## Conclusion

Network pharmacology and molecular docking help us predict potential compounds, targets, and pathways of mSMG against IR in T2DM and further guide experimental validation in vitro and in vivo, which conclude that mSMG and its representative compound berberine alleviate hepatic IR and promote glycogen synthesis, and its mechanism may involve the inhibition of TNF-α/JNK1/IRS-2 pathway. This study predicts the potential mechanism and displays scientific evidence for mSMG in the therapy of T2DM, which contributes to the drug development and may provide a new option for the complementary and alternative treatment of T2DM.

## Supplementary Information

Supplementary Material 1.

Supplementary Material 2.

## Data Availability

Data will be made available on request.

## Abbreviations

Akt: Protein kinase B
AUC: Area under the curve
DL: Drug-likeness
ELISA: Enzyme linked immunosorbent assay
FBG: Fasting blood glucose
FINS: Fasting insulin
GLUT: Glucose transporter
GO: Gene Ontology
GS: Glycogen synthase
GSK-3β: Glycogen synthase kinase-3β
HbA1c: Glycated hemoglobin
HFD: High-fat diet
HOMA: Homeostasis model assessment
IR: Insulin resistance
IRS: Insulin receptor substrate
JNK: C-Jun N-terminal kinase
KEGG: Kyoto Encyclopedia of Genes and Genomes
LDL-C: Low-density lipoprotein cholesterol
MAPK: Mitogen-activated protein kinase
NAFLD: Non-alcoholic fatty liver disease
NAS: NAFLD activity score
NS: Normal serum
OB: Oral bioavailability
OGTT: Oral glucose tolerance test
PI3K: Phosphatidylinositol 3 kinase
PPI: Protein–protein interaction
mSMG: Modified Si-Miao granule
mSMG-MS: MSMG-mediated serum
Ser: Serine
T2DM: Type 2 diabetes mellitus
TC: Total cholesterol
TCM: Traditional Chinese medicine
TCMSP: Traditional Chinese Medicine Systems Pharmacology database
TG: Triglyceride
TNF: Tumor necrosis factor
UPLC-Q-Orbitrap-MS: Ultra-high-performance Liquid Chromatography-Quadrupole-Orbitrap Mass Spectrometry
