# Correction: The improvement of modified Si-Miao granule on hepatic insulin resistance and glycogen synthesis in type 2 diabetes mellitus involves the inhibition of TNF-α/JNK1/IRS-2 pathway: network pharmacology, molecular docking, and experimental validation

**DOI:** 10.1186/s13020-024-01012-x

**Published:** 2024-10-21

**Authors:** Zebiao Cao, Xianzhe Wang, Zhili Zeng, Zhaojun Yang, Yuping Lin, Lu Sun, Qiyun Lu, Guanjie Fan

**Affiliations:** 1 State Key Laboratory of Dampness, Syndrome of Chinese Medicine, The Second Affiliated Hospital of Guangzhou University of Chinese Medicine & School of Basic Medical Sciences, Guangzhou University of Chinese Medicine, Guangzhou, China; 2 Department of Endocrinology, The Second Affiliated Hospital of Guangzhou University of Chinese Medicine, Guangzhou, China; 3 Postdoctoral Research Center, Guangdong Provincial Hospital of Chinese Medicine, Guangzhou, China; 4 Guangdong Provincial Academy of Chinese Medical Sciences, Guangzhou, China; 5 Guangzhou University of Chinese Medicine, Guangzhou, China; 6 Department of Breast Disease, The Second Affiliated Hospital of Guangzhou University of Chinese Medicine, Guangzhou, China; 7 School of Pharmaceutical Sciences, Guangzhou University of Chinese Medicine, Guangzhou, China

**Correction: Chinese Medicine (2024) 19:128** 10.1186/s13020-024-00997-9

Following publication of the original article [1], the authors reported that Fig. 2C and E were missing. The correct Fig. 2 has been provided in this correction.

**Fig. 2:**
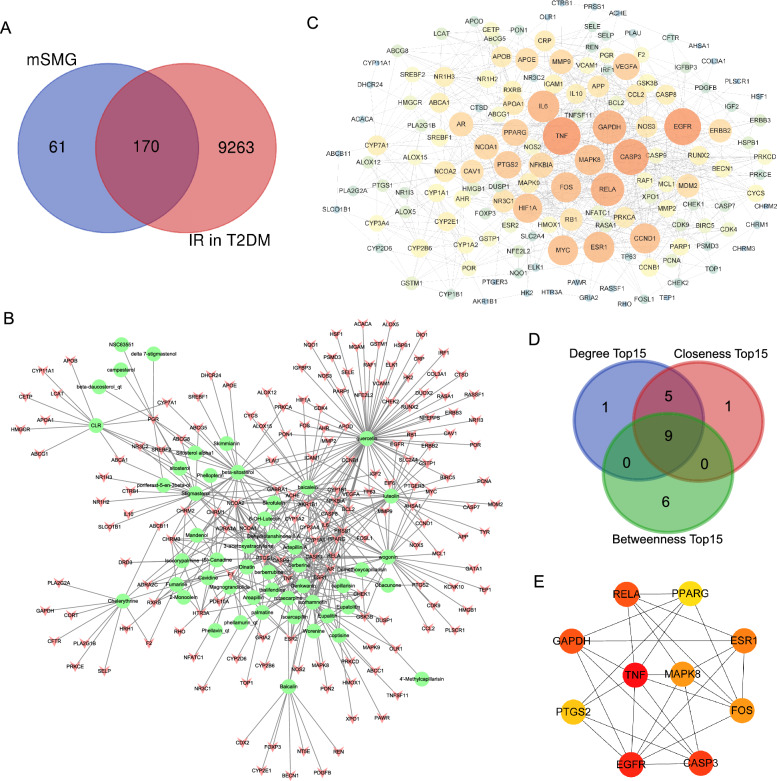
Potential action targets, compound-target network, PPI network, and hub targets of mSMG against IR in T2DM. **A** 170 intersecting targets between IR-associated genes in T2DM and candidate targets of mSMG were considered as the potential action targets of mSMG against IR in T2DM. **B** Compound-target network. A chartreuse ellipse represents a compound and a red “V” shape represents a target. **C** PPI network. A node represents a target. The size and the color of the node represents the value of the degree. Blue → yellow → red indicates that the degree value is from low to high, and the bigger the circle, the higher the degree value. **D** The intersecting 9 targets of the top 15 targets ranked by the 3 topological algorithms (Degree, Closeness, and Betweenness) were identified as the hub targets of mSMG against IR in T2DM. **E** PPI network of the 9 hub targets. The color of the node represents the value of the degree. Yellow → orange → red indicates that the degree value is from low to high

The original article [1] has been corrected.
