# Supplementary material for: The improvement of modified Si-Miao granule on hepatic insulin resistance and glycogen synthesis in type 2 diabetes mellitus involves the inhibition of TNF-α/JNK1/IRS-2 pathway: network pharmacology, molecular docking, and experimental validation
Source: Chin Med. 2024 Sep 16;19:128. doi: 10.1186/s13020-024-00997-9 (PMC11403785; doi:10.1186/s13020-024-00997-9)
Supplement: Supplementary file 1 — Supplementary Material 1. [file 13020_2024_997_MOESM1_ESM.docx]

Table S1 Information of 170 potential action targets of mSMG against IR in T2DM

| Target Symbol | Target full name |
| --- | --- |
| ABCA1 | ATP binding cassette subfamily A member 1 |
| ABCB11 | ATP binding cassette subfamily B member 11 |
| ABCC1 | ATP binding cassette subfamily C member 1 |
| ABCG1 | ATP binding cassette subfamily G member 1 |
| ABCG5 | ATP binding cassette subfamily G member 5 |
| ABCG8 | ATP binding cassette subfamily G member 8 |
| ACACA | Acetyl-CoA carboxylase 1 |
| ACHE | Acetylcholinesterase |
| ADRA1A | Alpha-1A adrenergic receptor |
| ADRA2C | Alpha-2C adrenergic receptor |
| AHR | Aryl hydrocarbon receptor |
| AHSA1 | Activator of 90 kDa heat shock protein ATPase homolog 1 |
| AKR1B1 | Aldose reductase |
| ALOX12 | arachidonate 12-lipoxygenase, 12S type |
| ALOX15 | arachidonate 15-lipoxygenase |
| ALOX5 | Arachidonate 5-lipoxygenase |
| APOA1 | apolipoprotein A1 |
| APOB | apolipoprotein B |
| APOD | Apolipoprotein D |
| APOE | apolipoprotein E |
| APP | Amyloid beta A4 protein |
| AR | Androgen receptor |
| BCL2 | Apoptosis regulator Bcl-2 |
| BECN1 | beclin 1 |
| BIRC5 | Baculoviral IAP repeat-containing protein 5 |
| CASP3 | Caspase-3 |
| CASP7 | Caspase-7 |
| CASP8 | Caspase-8 |
| CASP9 | Caspase-9 |
| CAV1 | Caveolin-1 |
| CCL2 | C-C motif chemokine ligand 2 |
| CCNB1 | G2/mitotic-specific cyclin-B1 |
| CCND1 | G1/S-specific cyclin-D1 |
| CDK4 | cyclin dependent kinase 4 |
| CDK9 | cyclin dependent kinase 9 |
| CDX2 | caudal type homeobox 2 |
| CETP | cholesteryl ester transfer protein |
| CFTR | CF transmembrane conductance regulator |
| CHEK1 | Serine/threonine-protein kinase Chk1 |
| CHEK2 | Serine/threonine-protein kinase Chk2 |
| CHRM1 | Muscarinic acetylcholine receptor M1 |
| CHRM2 | Muscarinic acetylcholine receptor M2 |
| CHRM3 | Muscarinic acetylcholine receptor M3 |
| COL3A1 | Collagen alpha-1(III) chain |
| CORT | cortistatin |
| CRP | C-reactive protein |
| CTRB1 | Chymotrypsinogen B |
| CTSD | Cathepsin D |
| CYCS | Cytochrome c |
| CYP11A1 | cytochrome P450 family 11 subfamily A member 1 |
| CYP1A1 | Cytochrome P450 1A1 |
| CYP1A2 | cytochrome P450 family 1 subfamily A member 2 |
| CYP1B1 | Cytochrome P450 1B1 |
| CYP2B6 | Cytochrome P450 2B6 |
| CYP2D6 | cytochrome P450 family 2 subfamily D member 6 |
| CYP2E1 | cytochrome P450 family 2 subfamily E member 1 |
| CYP3A4 | Cytochrome P450 3A4 |
| CYP7A1 | cytochrome P450 family 7 subfamily A member 1 |
| DHCR24 | 24-dehydrocholesterol reductase |
| DIO1 | Type I iodothyronine deiodinase |
| DRD3 | D(3) dopamine receptor |
| DUOX2 | Dual oxidase 2 |
| DUSP1 | dual specificity phosphatase 1 |
| EGFR | Epidermal growth factor receptor |
| EIF6 | Eukaryotic translation initiation factor 6 |
| ELK1 | ETS domain-containing protein Elk-1 |
| ERBB2 | Receptor tyrosine-protein kinase erbB-2 |
| ERBB3 | Receptor tyrosine-protein kinase erbB-3 |
| ESR1 | Estrogen receptor |
| ESR2 | Estrogen receptor beta |
| F2 | coagulation factor II, thrombin |
| F7 | Coagulation factor VII |
| FOS | Proto-oncogene c-Fos |
| FOSL1 | Fos-related antigen 1 |
| FOXP3 | forkhead box P3 |
| GABRA1 | Gamma-aminobutyric acid receptor subunit alpha-1 |
| GAPDH | glyceraldehyde-3-phosphate dehydrogenase |
| GATA1 | GATA binding protein 1 |
| GRIA2 | Glutamate receptor 2 |
| GSK3B | Glycogen synthase kinase-3 beta |
| GSTM1 | Glutathione S-transferase Mu 1 |
| GSTP1 | Glutathione S-transferase P |
| HIF1A | Hypoxia-inducible factor 1-alpha |
| HK2 | Hexokinase-2 |
| HMGB1 | high mobility group box 1 |
| HMGCR | 3-hydroxy-3-methylglutaryl-CoA reductase |
| HMOX1 | heme oxygenase 1 |
| HRH1 | histamine receptor H1 |
| HSF1 | Heat shock factor protein 1 |
| HSPB1 | Heat shock protein beta-1 |
| HTR3A | 5-hydroxytryptamine receptor 3A |
| ICAM1 | Intercellular adhesion molecule 1 |
| IGF2 | Insulin-like growth factor II |
| IGFBP3 | Insulin-like growth factor-binding protein 3 |
| IL10 | interleukin 10 |
| IL6 | Interleukin-6 |
| IRF1 | Interferon regulatory factor 1 |
| KCNK10 | potassium two pore domain channel subfamily K member 10 |
| LCAT | lecithin-cholesterol acyltransferase |
| MAPK8 | mitogen-activated protein kinase 8 |
| MAPK9 | mitogen-activated protein kinase 9 |
| MCL1 | Induced myeloid leukemia cell differentiation protein Mcl-1 |
| MDM2 | E3 ubiquitin-protein ligase Mdm2 |
| MGAM | Maltase-glucoamylase, intestinal |
| MMP2 | matrix metallopeptidase 2 |
| MMP9 | matrix metallopeptidase 9 |
| MYC | Myc proto-oncogene protein |
| NCOA1 | Nuclear receptor coactivator 1 |
| NCOA2 | Nuclear receptor coactivator 2 |
| NFATC1 | nuclear factor of activated T cells 1 |
| NFE2L2 | Nuclear factor erythroid 2-related factor 2 |
| NFKBIA | NF-kappa-B inhibitor alpha |
| NOS2 | nitric oxide synthase 2 |
| NOS3 | Nitric oxide synthase, endothelial |
| NOX5 | NADPH oxidase 5 |
| NPEPPS | Puromycin-sensitive aminopeptidase |
| NQO1 | NAD(P)H dehydrogenase [quinone] 1 |
| NR1H2 | nuclear receptor subfamily 1 group H member 2 |
| NR1H3 | nuclear receptor subfamily 1 group H member 3 |
| NR1I3 | Nuclear receptor subfamily 1 group I member 3 |
| NR3C1 | Glucocorticoid receptor |
| NR3C2 | Mineralocorticoid receptor |
| NT5E | 5'-nucleotidase ecto |
| OLR1 | Oxidized low-density lipoprotein receptor 1 |
| PARP1 | Poly [ADP-ribose] polymerase 1 |
| PAWR | pro-apoptotic WT1 regulator |
| PCNA | Proliferating cell nuclear antigen |
| PDE10A | cAMP and cAMP-inhibited cGMP 3',5'-cyclic phosphodiesterase 10A |
| PDGFB | platelet derived growth factor subunit B |
| PGR | Progesterone receptor |
| PLA2G1B | phospholipase A2 group IB |
| PLA2G2A | phospholipase A2 group IIA |
| PLAU | Urokinase-type plasminogen activator |
| PLSCR1 | phospholipid scramblase 1 |
| PON1 | Serum paraoxonase/arylesterase 1 |
| PON2 | paraoxonase 2 |
| POR | NADPH--cytochrome P450 reductase |
| PPARG | Peroxisome proliferator activated receptor gamma |
| PRKCA | Protein kinase C alpha type |
| PRKCD | protein kinase C delta |
| PRKCE | protein kinase C epsilon |
| PRSS1 | Trypsin-1 |
| PSMD3 | 26S proteasome non-ATPase regulatory subunit 3 |
| PTGER3 | Prostaglandin E2 receptor EP3 subtype |
| PTGS1 | Prostaglandin G/H synthase 1 |
| PTGS2 | prostaglandin-endoperoxide synthase 2 |
| RAF1 | RAF proto-oncogene serine/threonine-protein kinase |
| RASA1 | Ras GTPase-activating protein 1 |
| RASSF1 | Ras association domain-containing protein 1 |
| RB1 | Retinoblastoma-associated protein |
| RELA | Transcription factor p65 |
| REN | renin |
| RHO | Rhodopsin |
| RUNX2 | Runt-related transcription factor 2 |
| RXRB | Retinoic acid receptor RXR-beta |
| SELE | E-selectin |
| SELP | selectin P |
| SLC2A4 | Solute carrier family 2, facilitated glucose transporter member 4 |
| SLCO1B1 | solute carrier organic anion transporter family member 1B1 |
| SREBF1 | sterol regulatory element binding transcription factor 1 |
| SREBF2 | sterol regulatory element binding transcription factor 2 |
| TEP1 | Telomerase protein component 1 |
| TNF | tumor necrosis factor |
| TNFSF11 | TNF superfamily member 11 |
| TOP1 | DNA topoisomerase I |
| TP63 | Cellular tumor antigen p53 |
| TYR | Tyrosinase |
| VCAM1 | Vascular cell adhesion protein 1 |
| VEGFA | Vascular endothelial growth factor A |
| XPO1 | exportin 1 |
