# Supplementary material for: The improvement of modified Si-Miao granule on hepatic insulin resistance and glycogen synthesis in type 2 diabetes mellitus involves the inhibition of TNF-α/JNK1/IRS-2 pathway: network pharmacology, molecular docking, and experimental validation
Source: Chin Med. 2024 Sep 16;19:128. doi: 10.1186/s13020-024-00997-9 (PMC11403785; doi:10.1186/s13020-024-00997-9)
Supplement: Supplementary file 2 — Supplementary Material 2. [file 13020_2024_997_MOESM2_ESM.docx]

Table S2 30 compounds of mSMG identified by UPLC-Q-Orbitrap-MS

| Peak | Compound | RT[min] | Formula | CAS | Structure |
| --- | --- | --- | --- | --- | --- |
| 1 | DL-Arginine | 0.99 | C6H14N4O2 | 7200-25-1 | 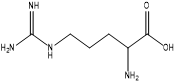 |
| 2 | L-Glutamic acid | 1.31 | C5H9NO4 | 56-86-0 | 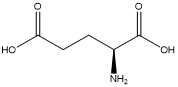 |
| 3 | 2-Hydroxyglutaric acid | 1.62 | C5H8O5 | 2889-31-8 | 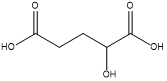 |
| 4 | Pipecolic acid | 1.82 | C6H11NO2 | 535-75-1 | 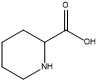 |
| 5 | Arcapillin | 2.13 | C18H16O8 | 83162-82-7 | 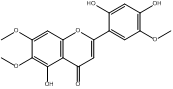 |
| 6 | 7-Hydroxycoumarine | 8.24 | C9H6O3 | 93-35-6 | 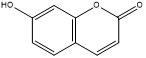 |
| 7 | baicalein | 8.50 | C15H10O5 | 491-67-8 | 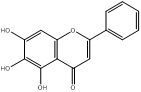 |
| 8 | wogonin | 9.26 | C16H12O5 | 632-85-9 | 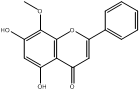 |
| 9 | Asperulosidic acid | 9.93 | C18H24O12 | 25368-11-0 | 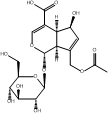 |
| 10 | luteolin | 10.03 | C15H10O6 | 491-70-3 | 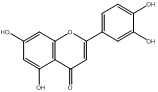 |
| 11 | Phellamurin | 12.50 | C26H30O11 | 52589-11-4 | 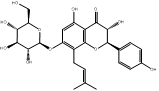 |
| 12 | Caffeic acid | 12.76 | C9H8O4 | 331-39-5 | 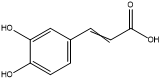 |
| 13 | Kaempferol | 13.58 | C15H10O6 | 520-18-3 | 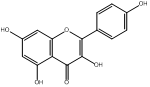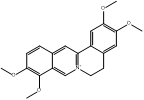 |
| 14 | palmatine | 15.77 | C21H22NO4+ | 3486-67-7 |  |
| 15 | quercetin | 15.87 | C15H10O7 | 117-39-5 | 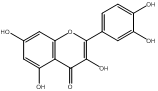 |
| 16 | coptisine | 16.34 | C19H14NO4+ | 3486-66-6 | 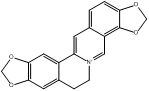 |
| 17 | Sedanolide | 16.44 | C12H18O2 | 6415-59-4 | 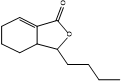 |
| 18 | berberine | 17.99 | C20H18NO4+ | 2086-83-1 | 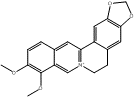 |
| 19 | Cafestol | 18.93 | C20H28O3 | 469-83-0 | 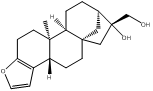 |
| 20 | isorhamnetin | 19.61 | C16H12O7 | 480-19-3 | 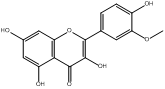 |
| 21 | D-(-)-Mannitol | 1.33 | C6H14O6 | 69-65-8 | 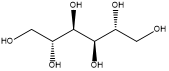 |
| 22 | Gluconic acid | 1.4 | C6H12O7 | 526-95-4 | 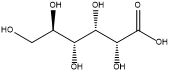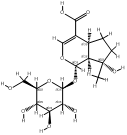 |
| 23 | Mussaenosidic acid | 8.22 | C16H24O10 | 82451-22-7 |  |
| 24 | Esculin | 8.83 | C15H16O9 | 531-75-9 | 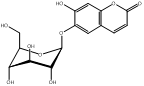 |
| 25 | 5-Sulfosalicylic acid | 9.85 | C7H6O6S | 5965-83-3 | 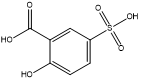 |
| 26 | Vanillin | 10.67 | C8H8O3 | 121-33-5 | 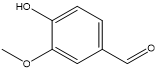 |
| 27 | Chlorogenic acid | 11.28 | C16H18O9 | 327-97-9 | 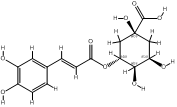 |
| 28 | Atractylodin | 21.94 | C13H10O | 55290-63-6 |  |
| 29 | Linoleic acid | 21.94 | C18H32O2 | 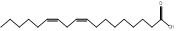60-33-3 |  |
| 30 | TOFA | 23.05 | C19H32O4 | 54857-86-2 | 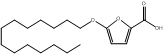 |
